# Substance Abuse, Relapse, and Treatment Program Evaluation in Malaysia: Perspective of Rehab Patients and Staff Using the Mixed Method Approach

**DOI:** 10.3389/fpsyt.2016.00090

**Published:** 2016-05-26

**Authors:** Qiu Ting Chie, Cai Lian Tam, Gregory Bonn, Hoang Minh Dang, Rozainee Khairuddin

**Affiliations:** ^1^Jeffery Cheah School of Medicine and Health Sciences, Monash University Malaysia, Bandar Sunway, Malaysia; ^2^International Research Fellow of the Japan Society for the Promotion of Science, Graduate School of Education and Human Development, Nagoya University, Nagoya, Japan; ^3^Centre for Research, Information and Service in Psychology (CRISP), Vietnam National University, Hanoi, Vietnam; ^4^Faculty of Social Science and Humanities, Psychology and Human Development, National University of Malaysia (UKM), Bangi, Malaysia

**Keywords:** substance abuse, motivation to change, patient satisfaction, staff perception, treatment evaluation

## Abstract

This study examined reasons for substance abuse and evaluated the effectiveness of substance treatment programs in Malaysia through interviews with rehab patients and staff. Substance rehab patients (aged 18–69 years; *n* = 30) and staff (ages 30–72 years; *n* = 10) participated in semi-structured interviews covering a range of topics, including family and peer relationships, substance use and treatment history, factors for substance use and relapse, motivation for entering treatment, work experience, job satisfaction, treatment evaluation, and patient satisfaction. Most patients did not demonstrate the substance progression trend and had normal family relationships. Most patients reported having peers from normal family backgrounds as well. Various environmental and personal factors was cited as contributing to substance abuse and relapse. There was no significant difference between patient and staff program evaluation scores although the mean score for patients was lower. A holistic treatment approach with a combination of cognitive–behavioral, medical, social, and spiritual components was favored by patients. Suggestions for improving existing programs include better tailoring treatment to individual needs, and providing more post-treatment group support.

## Introduction

On a global level, substance abuse continues to be a longstanding public health issue (1). In Malaysia, although there have been periods of declining arrests and admissions to rehab centers (2), the most recently reported statistics in 2013 show significant increases in the number of admissions to rehab centers as well as relapses among reformed substance abusers. The reasons for these changes are not immediately clear. There is insufficient evidence within the Malaysian context to indicate that the increase in treatment admissions are the direct result of increased enforcement activity and subsequent increase in legal referrals, or due to changes in treatment admission policies, whereby substance users voluntarily register for treatment without fear of prosecution. Past studies conducted within Western settings were also more likely to research compulsory and voluntary treatment of substance abuse in association with readiness to change and treatment effectiveness (retention rates or post-treatment outcomes) or the relationship between coercion and compulsory treatment (3, 4). However, the United Nations (UN) suggests that globally, about one in six problem substance users receive treatment each year (1), so generally we can assume that the levels of substance abuse in Malaysia have not declined significantly overall.

Patterns of substance abuse are generally categorized into poly-substance or mono-substance (5). The World Health Organization defines poly-substance abuse as the concurrent (taken at the same time) or sequential (one substance taken, followed by another) abuse of more than one substance or type of substance, with dependence upon at least one (6). By contrast, mono-substance abuse is defined as the exclusive use of one substance, which is, in fact, a rare occurrence (7). In the past, some studies suggested a progression in substance use from *soft* (substances that do not cause physical addiction but may lead to a psychological dependence) to *hard* drugs (substances that lead to physical addiction) (8, 9). Others, however, such as Peele and Brodsky (10) have dismissed this theory as a cultural myth. Coffield and Gofton (11) similarly found that most users did not categorize substance use as a progression from soft to hard. Although, the use of “soft” drugs, such as marijuana, was highly correlated to “hard” drugs, such as heroin and cocaine, the relationship showed few signs of causality (11). Overall, this issue still seems to be open to debate (12).

Western literature on the contributory factors of substance abuse and relapse commonly points to family factors and peer influence. Family factors identified as significant predictors of substance abuse include: parental substance abuse (13), chaotic and unsupportive family conditions (14), strength of the parent–child relationship (15), and being raised in a single-parent family or an adopted family (16). On the other hand, open parent–child communication about substance abuse and positive parent–child relationships (17) were seen as important protective factors, especially among African-American adolescents.

Similarly, surveys of substance users in Malaysia commonly cite peer influence as the top reason for initiating or relapsing into substance use (2). Especially during the formative years of adolescence, peers influence others’ behavior through constant association, and reinforcement (18–20). Often adolescents engage in substance use due to the social pressure to belong and be accepted by their peer group (20), and to conform to group identities such as pop, techno, hippie, and skate or hip-hop groups, which are often linked to use of marijuana and other substances (21, 22). Such influences, however, may be moderated by individual factors, such as assertiveness, where findings related to assertiveness and substance use generally show that substance addicts and court-referred patients tend to be less assertive, less socially assertive, and more socially anxious than non-user populations (23). Thus, more assertive personalities are probably better able to resist peer pressure than others.

In a similar vein, studies of substance rehab patients in Peninsular Malaysia found that self-confidence, social support, and family support were key protective factors against relapse (24). This relates closely to previous research that has divided drug abuse predictors into the broad categories of personality (e.g., depression and anti-social behavior) and environment (e.g., family dysfunctions and unemployment)-related factors (25). Thus, depending upon the contributing factors for each individual, different types of treatment approaches are expected to be more effective. For example, among those with limited job skills, provision of vocational training in rehab centers could help to avoid unemployment-related relapse (a social factor) (25). Others with more psychologically related issues, such as depression or anxiety, would obviously benefit from different approaches, psychodynamic and/or pharmaceutical, which could help alleviate their desire to self-medicate.

In regard to treatment availability and accessibility in Malaysia, an upgrading exercise was recently carried out by Malaysia’s National Anti-Drugs Agency (NADA) whereby existing rehab centers were reclassified into a more systematic and specialized structure. Thus, a number of categories of facilities, such as Cure and Care 1Malaysia clinic, Cure and Care Rehabilitation Center (CCRC), and Cure and Care Service Centers (CCSC), were created with somewhat different sets of specialized treatment offerings (26). Even with more specialized offerings, however, low treatment motivation among rehab patients and treatment compliance problems remain major obstacles. An obvious contributor to this is the fact that a majority of substance users currently under rehabilitation in Malaysia are doing so under court orders (2). Thus, even with somewhat improved treatment options, self-motivation to change remains an important challenge (27). In most cases, most substance users become motivated to induce change and seek treatment after experiencing severe implications of substance use, such as the sense powerless or a drop in self-image and self-esteem (28).

Although the assessment of patient satisfaction with treatment is an essential element in evaluating health-care quality and service provision (29), the use of patient satisfaction measures to assess treatment process and outcomes for substance abuse patients has been limited (30). This may stem from a belief that patient satisfaction is secondary to the counseling relationship (31). Moreover, most evaluation studies have been conducted in Western settings with comparatively limited done in Asia and almost none in Malaysia. Overall, this literature has yielded limited and inconsistent findings (32). For instance, a study in Illinois found that matched service needs were associated with the perception that treatment had helped patients to control substance use but not associated with reduced substance use (33). Another study in California found positive relationships between service intensity and patient satisfaction with treatment (34). A separate study of the association between patient satisfaction and treatment outcomes, which was conducted in the United States, revealed that favorable patient satisfaction evaluations of treatment nearing the time of discharge had a positive net effect on primary and overall substance use outcomes at 1-year post-treatment (32). This result was independent of the measured effects of predictors like treatment duration, counseling hours/intensity, agreement and adherence to treatment goals, and baseline substance use. Naturally, patients who adhered and agreed with treatment goals had significantly better improvement in substance use outcomes.

Nearly all of these previous studies just looked at the views of substance users, although they were recruited from different settings (35). To date, there is limited research focusing on primary treatment staff attitudes or beliefs regarding patients’ substance use (36). Thus, this study aimed to examine beliefs about substance abuse and relapse from the perspectives of both rehab patients and staff as well as to obtain feedback from patients and staff about the quality of substance treatment services in Malaysia. Interview data were evaluated using a Grounded Theory (social constructivist perspective) approach. Thus, although the initial questions were guided by some of the issues mentioned in the preceding review, no specific hypotheses were formed prior to the research. Employing the concept of iterative inquiry, we chose to progressively develop our enquiries and aim our interpretations at accessing the phenomenological experiences of participants. Nevertheless, our iterative inquiry focused on addressing the following research questions:

(a) What user demographics would emerge in regard to substance use progression, and conditions of family and peer relationships?
(b) Based on rehab patients’ self-evaluation, what levels of assertiveness against substances would patients exhibit at the point of treatment?
(c) What themes would emerge from patient and staff interviews in relation to substance use and relapse factors, as well as motivation to seek treatment? What similarities and differences would emerge from these themes?
(d) Are there significant differences in treatment satisfaction scores between patients and rehab staff?
(e) From the perspective of patients and staff, which treatment components are favorable and what are the limitations? What improvements would be suggested by both parties to increase treatment effectiveness?

## Materials and Methods

### Ethics

Before the commencement of data collection, ethics approval (CF13/511 – 2013000227) was sought and received from the Monash University Human Research Ethics Committee (MUHREC). Stringent review was conducted in refining the study materials, protocol, and methods to protect the confidentiality of responses provided by rehab patients and staff.

### Sampling and Recruitment Criteria

Purposive sampling was used to recruit participants because strategic choices on the participants that should be included in the sample had to be made based on research criteria, such as specialist knowledge, the capacity and willingness to participate, the ability for legal consent, and unique perspective on the research issue (37). Participants must be rehab patients and staff above the age of 18 years. The patients must have stayed at the current rehab center for at least 3 months, while the rehab staff must have had at least 6 months of working experience. Additionally, patients who were medically ill, suffering from the side effects of substance use or physically injured were excluded from participation.

### Participants

#### Group 1: Substance Rehab Patients

##### Age Group

In total, there were 30 patients involved in this research. Fifteen patients were from a government rehab center located in Klang (an urban area in the state of Selangor, Malaysia), while another 15 were from a private rehab center (located in Hulu Langat, a mixed urban–rural area of Selangor). The age group comparison found that a higher proportion of patients in the private center were between 30 and 39 years old (46.7%), while a majority of patients in the government center were from the younger population between the age of 20 and 29 years old (26.7%). This was followed by the 50- to 59-year-olds (20.0%) for the government center and the 40- to 49-year-olds (33.3%) for the private center. There were 13.33% of government patients under the age of 20 years, while there were no patients from this age group admitted to the private center.

##### Ethnic Composition

There were differences in the ethnic composition of patients with Malays (12 patients) comprising the majority in the government center, followed by patients of Indian ethnicity (2 patients), and one Chinese rehab patient. By contrast, the Chinese (nine patients) made up the highest proportion of patients in the private center, followed by patients of Indian ethnicity (five patients) and one rehab patient from the ethnic Lun Bawang in Sarawak.

##### Marital Status

The majority of government and private patients were single (73.3%, respectively). Precisely 26.7% of government patients were married and equal proportions of private patients were married (13.3%) or divorced (13.3%).

##### Educational Status

A majority of patients in both government (53.3%) and private (66.7%) centers were educated, as they had completed at least an upper secondary level of education. In fact, 6.7% of government patients and private patients, respectively, had a graduate diploma or underwent pre-university education. Precisely 20.0% of government patients and 13.3% of private patients had at least a lower secondary level education. In both patient groups, 13.3%, respectively, had obtained at least a primary school level education although there were 6.7% of private patients who had never undergone formal schooling at all.

##### Age of Initiation

The mean age of initiating substance use among government patients was 18.87 years (SD = 4.34), while the mean age for private patients was slightly lower at 18.60 years (SD = 4.36).

##### Substance Use History

An investigation of the presence of a progression pattern in substance use (from soft to hard substances) by examining the substance types used by patients prior to treatment admission revealed that overall, a slightly higher proportion of rehab patients (*n* = 15, 50.0%) did not demonstrate the progression trend as compared to those who did (*n* = 13, 43.3%). Partial progression (involves oscillating from soft to hard substances, and the subsequent use of a substance that contained both soft and hard drug properties) was demonstrated by two patients (6.7%). Analysis of substance use patterns revealed that a majority of government patients were poly-substance users (*n* = 12), with only three mono-substance users. On the other hand, there were eight poly-substance users and seven mono-substance users in the private center. There was a wider period of addiction for government patients between 3 months and 40 years. The addiction range for private patients was between 5 and 33 years.

##### Parents’ Occupation

Patients in both rehab centers came from middle-class working families. Most patients in the government and private center have mothers who were housewives (*n* = 10; 66.7%, respectively). Among private patients, there were 2 (13.3%) mothers who worked as business assistants. On the other hand, a higher proportion of fathers belonging to government patients were lorry drivers (*n* = 2, 13.3%) and government servants (*n* = 2, 13.3%). One government patient was unable to provide this information as his father had left the family when he was a child. Among private patients, most fathers were businessmen (*n* = 4, 26.7%) and civil servants (*n* = 2, 13.3%).

### Past Rehabilitation Experience

A higher proportion of rehab patients in the government (*n* = 9, 60.0%) and private (*n* = 8, 53.3%) center have no treatment history before entering the current rehabilitation. By contrast, six government patients (40.0%) and seven private patients (46.7%) reported having relapsed and have received treatment from other rehab centers in the past.

#### Group 2: Substance Rehab Staff

##### Age Group

Analysis of the age group distribution revealed a wider age group among staff in the government center, with a majority of them (60.0%) in the 30–39 years age group. Equal proportions of staff followed this in the 40–49 years (20.0%) and 50–59 years age group (20.0%). In comparison, most staff in the private center were in the senior age group with 80.0% of staff between the age 40 and 49 years and another 20.0% in the 70 and 79 years age group.

##### Educational Status

Staff from the government center possessed higher learning qualifications with 80.0% having a graduate diploma and 20.0% who pursued graduate degree studies. This was in contrast with the staff in the private center, whose highest level of qualification was lower secondary schooling (60.0%) and primary level education (40.0%).

##### Work Experience

The job scope for staff in both rehab centers involved administrative work and direct-contact work with patients (e.g., counseling, vocational training, and spiritual studies). Staff from private center had longer working experience as compared to staff in the government center. The private staff had an average of 12.8 years (SD = 7.33) of total working experience and 11.4 years (SD = 6.88) of direct-contact experience. On the other hand, staff in the government center had an average of 4.9 years (SD = 3.17) in both total working experience and direct-contact experience.

##### Materials

###### Patient Interview Checklist

Items in the interview checklist were adapted from several resources (38–42). The topics explored in the patient interview are (a) demographics: family background, age group, gender, ethnicity, and educational status, (b) substance history, (c) contributory factors of substance abuse and relapse, (d) admission to center history, (e) evaluation of treatment, and (f) suggestions for improving treatment. Six items from the Assertion Questionnaire in Drug Use (AQ-D) by Callner and Ross (41) that focused on assertion in male heavy substance users were used. The AQ-D has demonstrated good reliability and excellent concurrent validity.

Family relationships among substance users and their peers were examined using a 30-item scale whereby patients had to rate their responses on a 4-point scale (1 = Not at all true to 4 = Very true). The minimum score for the relationship with parents scale was 20, while the maximum score was 80. Higher scores indicated problematic relationship with parents. The minimum score for the scale investigating family relationships among the peers was 10, while the maximum score was 40. Higher scores demonstrated more behavior problems among peers (41). The clinical cut-off score for the family relationship scale was 37.01 and peer relationship was 26.66.

A Session Evaluation Questionnaire (SEQ) by Stiles and Snow (42) was included to measure the impact of clinical sessions on patients’ feelings and current emotions, at the point of interview. Patients’ perceptions were measured using two dimensions: depth and smoothness. The post-session mood was measured using another two dimensions: positivity and arousal. Depth refers to a session’s perceived power and value, while Smoothness refers to the comfort, relaxation, and pleasantness felt during the session. Positivity refers to feelings of confidence, clarity, and happiness, while Arousal refers to active and excited feelings as opposed to calm and quiet. The four dimensions were scored separately and the total scores were the sum of item ratings. The SEQ has good internal consistency.

###### Staff Interview Checklist

The interview topics cover (a) demographics: family background, age group, gender, ethnicity, and educational status, (b) working experience, (c) perceptions of reasons for substance use and relapse, (d) perspective on reasons for substance abusers entering rehab, and (e) satisfaction with work and the rehab program.

##### Procedure

An official letter requesting permission to conduct interviews with substance rehab patients and staff was submitted to the Policy Planning and Research Department, Malaysian NADA. Permission to conduct interviews [Ref No: ADK 60/1/7, Vol. 12(81)] was granted in a government CCSC and a private rehabilitative center in the state of Selangor, Malaysia. With permission from the center administrators, posters were placed on notice boards within the center grounds and flyers were situated in administrative offices. Patients and staff who were interested could register in a form. The center administrators played an important role in aiding the selection process by identifying patients who were medically fit to participate.

Thirty patients (15 each from the government and private center) and 10 staff (5 each from the government and private center) were eligible to participate and completed the interviews. A group briefing was conducted prior to the interviews to ensure that participants were aware of the nature of the study, their rights to withdraw without consequences, and measures to protect participant confidentiality in terms of the written report and subsequent publication of findings. It was repeatedly emphasized to the participants during the interviews that participation was voluntary and would not affect treatment or job benefits received at the center. Before the commencement of the interviews, informed consent was obtained from all participants. The interview sessions were conducted one-to-one within 3 months. The interview responses were recorded word-by-word, by hand, due to ethical concerns of breach in patient confidentiality with audio recordings. In addition to the interview responses, field notes from observations were also coded and analyzed for similar and unique themes.

### Data Analysis

SPSS version 20 was used to perform descriptive statistics and *t*-tests, while qualitative data were managed and analyzed using NVivo 10. We applied three principles in Grounded Theory (social constructivist perspective) during data collection, management, and analysis. These principles include the use of an interpretative approach, with flexible guidelines and emphasis on the values, views, beliefs, feelings, and assumptions of individuals; an active role by the researcher during the collection and coding of rich data for analysis, as well as the inclusion of the researcher’s personal values, beliefs, and experiences to the data (43).

Thematic analysis was used to identify, analyze, and report themes within qualitative data, with Braun and Clarke’s six phases of thematic analysis as a reference (44). Data familiarization was achieved by reading it repeatedly and generating initial ideas. Initial codes were generated by coding data systematically. Potential codes were subsequently collated into general themes, which were reviewed through thematic mapping. The themes were defined by generating concepts and thematic names before the final step, in which a report is produced with selected examples and relating themes with the research question.

## Results

### Quantitative Analysis

#### Patients’ Assertiveness

Analysis of assertion scores revealed that 14 patients (46.7%) reported being assertive to offers of substances by their friends or strangers in a social party. This was followed by 10 patients (33.3%) who reported being extremely non-assertive when faced with offers of substances. Only six patients (20.0%) felt they were extremely assertive in resisting substances offered by strangers or preventing friends from bringing substances to their home. All patients revealed that they would only use substances out of home, as they did not want their family to know about their substance use.

#### Family Relationships

The analysis demonstrated that a majority of the patients (*n* = 25, 83.3%) reported having normal relationships with their family, indicating that most patients reported having close relationships with their parents and reported receiving parental love and support. The family environment was also reported as harmonious, with constant communication between family members. A higher proportion of patients also came from families with stable incomes and had parents with no history of substance abuse or behavior problems. Only five patients (16.7%) had family relationships that were classified under clinical levels. These patients reported having conflicts with a step-parent, experienced lack of recognition and trust from their family members due to a past mistake, and felt that there were no clear boundaries and guidance set within their family structure.

Similarly, most patients (*n* = 25, 83.3%) reported having friends from normal family backgrounds, whereby they were not isolated or suffered rejection and hostility from their parents. Moreover, most of their peers did not demonstrate clinical levels of rebelliousness and problem behaviors, such as getting involved in fights, stealing, and robbery. Only five patients (16.7%) reported having peers, who engaged or showed favorable attitudes toward problem behaviors and had families that demonstrated distant and isolated relationships. However, all patients agreed that the influence of peers in substance use were irrefutable in their respective circumstances.

#### Patient Satisfaction with Treatment

As presented in Table 1, most patients (93.3%) perceived that their most recent session was deep in content. Only one patient felt that the contents touched the surface in resolving problems faced by substance users and were not as useful. Another patient felt that the session was in-depth occasionally and, thus, was able to learn some skills to resolve personal issues. However, there were times where problem-solving techniques discussed were not as useful in aiding the resolution of substance use issues.

**Table 1:** **Drug rehab patients’ session evaluation by dimension**.

Dimensions of SEQ	Patients (*N* = 30)
**Depth**	
Shallow	1 (3.3%)
Neither shallow nor deep	1 (3.3%)
Deep	28 (93.3%)
**Smoothness**	
Rough	1 (3.3%)
Neither rough nor smooth	1 (3.3%)
Smooth	28 (93.3%)
**Positivity**	
Negative	1 (3.3%)
Positive	29 (96.7%)
**Arousal**	
Peaceful	11 (36.7%)
Neither peaceful nor aroused	1 (3.3%)
Aroused	18 (60.0%)

Most patients (93.3%) reported that the sessions were well conducted, pleasant and easy to understand and proceeded smoothly to schedule. Only one outpatient reported feeling hassled with last-minute changes to the program. Another patient felt that the treatment sessions were relatively smooth with minor glitches, when the counselors did not arrive for their scheduled sessions or when activities were canceled.

Most patients (96.7%) also felt the sessions provided positive messages and were pleased that the staff and counselors were friendly and encouraging. After the session, they were more focused and confident about working toward their goal of overcoming substance use. Only one patient was rather negative about his experience undergoing treatment. This patient entered treatment at an old age and reportedly received treatment to be substance-free and to live more comfortably in his remaining lifespan.

Arousal was another dimension that measured post-session mood of patients. Precisely 60.0% of patients reported feeling excited and empowered at the end of the session. This was followed by 36.7% of patients, who felt peaceful but a little excited on the prospect of undertaking treatment tasks. Only one patient felt neither excited nor particularly motivated to accomplish his tasks.

#### Differences in Patient and Staff Program Evaluation Scores

A *t*-test comparison between program evaluation scores by rehab patients and staff yielded higher mean scores for staff ($\bar{x}=8.10$, SD = 2.18) as compared to patients ($\bar{x}=7.27$, SD = 2.18). Nevertheless, there was no significant difference between the patients and staff scores [*t* (39) = −1.046, *p* > 0.05].

### Qualitative Analysis

#### Factors for Substance Abuse

There were two broad themes generated in relation to contributory factors for substance abuse in patients and staff responses, which are environmental and personal factors (see Table 2). *Peer influence* (38 references) and *family conflicts* (11 references) were viewed by both rehab patients and staff as common external influences that led to substance use. *Curiosity* (35 references), *tension release* (17 references), *enjoyment* (8 references), *relationship and health issues* (8 references), and *unemployment* (6 references) were viewed as common personal factors leading to substance use. The dual role of unemployment as a predictor and outcome of substance abuse was clearly indicated in the patients’ responses, whereby substances was used as a past-time activity while unemployed (predictor) and substance use resulted in the inability to maintain employment (outcome).

**Table 2:** **Factors for drug abuse, relapse, and motivation to seek treatment from the perspective of rehab patients and staff**.

Categories	Themes	Sub-themes	Quotes
Initiation of drug use	Environmental factors	Peer influence	*“The first reason was my friends’ influence. They let me taste it and when I found that I like it, I started to search for more on my own.” [GP15]*
			*“Peer influence is the most common reason as most youths are easily influenced by friends in Year 6 or Form 3.” [GS04]*
		Family conflict	*“There were also a lot of family issues and conflicts. At that time, my mother had passed away and I felt that there was no one around to love and care for me. Even my siblings did not want to talk to me.” [PP03]*
			*“Family conflicts with my step-mother also sort of contributed to my habit though I have a good relationship with my father.” [GP01]*
	Personal factors	Curiosity	*“It was also about satisfying my curiosity about drugs. At that time, everyone around me was doing drugs.” [PP02]*
			*“Mostly curiosity and because of their friends…” [GS05]*
		Enjoyment	*“My friends and I started taking drugs for the enjoyment during happy hour at the pub.” [PP06]*
		Tension release	*“I often felt anxious and also took drugs as part of my method of dealing with all the tension.” [PP03]*
			*“Using drugs as a way to release tension is skewed towards the highly educated group.” [GS01]*
		Relationship issues	*“…I met a girl I liked while I was working and we got engaged. But not long after, the engagement was broken off.” [GP01]*
		Health issues	*“…I was sick and experiencing a lot of physical pain. I was using drugs as a painkiller but it didn’t work. Instead, it made the pain worse.” [PP06]*
		Unemployment	*“In between jobs, I sometimes find myself unemployed and I took drugs to fill time.” [PP01]*
			*“Unemployment was not a contributing factor to drug use but rather an effect of taking drugs. When I was under the influence of drugs, it was hard to concentrate on finding and maintaining a job.” [PP03]*
Drug relapse	External factor	Family conflicts	*“I was really unhappy and stressed at that time due to many family conflicts occurring.” [PP06]*
			*“Family issues and conflicts were more often the reason for relapse rather than initiation of drug use.” [GS02]*
		Old peer influence	*“There was also the influence of friends from the old group and workplace.” [GP02]*
			*“It was no longer due to influence of friends because most of my friends are no longer around. Most of them have died because of drugs.” [PP09]*
			*“…former inmates tend to fall back to old habits when there is strong external influence from friends.” [GS02]*
	Personal factor	Drug urges	*“I couldn’t stand the urge and memories of taking drugs.” [GP15]*
			*“…once they are released back into society, most of them are unable to stop their urge towards drugs when they fall back into the same group of friends.” [GS01]*
Motivation for treatment	Impact on self	Financial constraints	*“I could not stand it physically, emotionally and financially anymore. I had to find enough money to get my supply. Moreover, I am getting older.” [GP04]*
			*“I was financially unable to support my drug habit anymore. My money was only sufficient to live day by day.” [GP07]*
	Impact on family	Inability to support aging parents	*“The thing that drives me to stop is that I am unable to take care and support my aging parents. Money that should be contributed to the family was being used to buy drugs instead. So this cannot go on.” [GP15]*
		Neglected responsibilities	*“My family was the primary motivation. My habit was causing me to neglect them.” [PP06]*
	Impact on work		*“…drugs were affecting my work and were causing family conflicts.” [GP02]*
	Impact on health	Physical deterioration	*“My family encouraged me to stop as my health was not so good. I also wanted to change for the future and myself.” [PP05]*
			*“My health was getting bad as I got older. I couldn’t stand it anymore.” [PP09]*
		Emotional torture	*“…the difficulties of undergoing the constant mental torture.” [GP14]*
	Personal wish to heal	Disappointed with past life	*“Mainly, I was disappointed with my life and I wanted to change for the better on my own.” [PP08]*
		Wanting a normal life	*“I wanted to change. After being caught and entering prison, I can’t have a normal life anymore. I can’t get married and my parents are getting old.” [PP12]*
		Wishing a better future	*“I didn’t like that kind of lifestyle anymore. I felt that it was time I stopped and heal myself from this habit.” [GP05]*
	Religious guidance		*“I have been involved with drugs for so long that I feel very tired. I have no proper work, and I feel the rejection from my family and society. So, I asked God to help me and the chance to help myself was given.” [PP03]*
			*“After I came out of prison, I did not have anywhere to go. So I relapsed after falling back with the old crowd of friends. A pastor whom I knew advised me to enter rehabilitation.” [PP14]*
	Court orders		*“To be honest, I entered the center without thoughts of stopping. I got caught and was sent to rehab through court orders.” [GP01]*

In addition to these, five sub-themes were found specifically in staff responses: *media influence* (environmental factor), *confidence issues*, *educational status*, *lack of religious guidance*, and *energy boost* (personal factors). The role of *media influence* was credited by a staff as instrumental toward encouraging substance use among younger substance users. It was viewed that the wide exposure to internet and television was responsible for young people engaging in drug use to satisfy curiosity about illicit substances.

A staff also cited *confidence issues* as a contributory factor of substance use. It was viewed that some individuals used substances to increase levels of confidence during socialization or in stressful situations. However, there were individuals who became addicted due to overconfidence in their ability to stop using substances whenever they wanted. There were contradictory opinions about *educational status* as a reason for substance abuse. However, two staff elaborated that there is a public perception that substance abuse is more commonly associated with individuals of lower educational status. While it was acknowledged that about 65% of drug addicts did not complete their education and did not have proper role models with the family or school to guide them, there has been an increase in number of substance users from highly educated backgrounds in recent years. Additionally, a staff viewed a *lack of religious guidance* (personal factor) as a driving factor. It was perceived that the individual is driven to use substances when they lose their direction in life and they will cope well by following religious teachings. *Energy boost* was also cited by a staff, as an important factor for substance use among students to cope with academic and social stress. Furthermore, an increasing number of students were reportedly using stimulants to improve academic performance by studying for days without sleep.

#### Factors for Substance Relapse

Themes under environmental and personal factors were also found in responses about substance relapse factors. *Peer influence from old friends* (eight references) within the neighborhood and workplace and *family conflicts* (two references) were the main external factors cited by patients and staff (see Table 2). However, old peer influence was not significant for elderly substance users as most of their peers were no longer present due to old age or the effects of substance use. Patients and staff also agreed that the inability to withstand *drug urges* (10 references) after re-entering society was also a personal factor for relapse. This factor was closely associated with old peer influence. In addition, the temptation to use substances reportedly re-emerged when patients attempt to cope with life and societal pressures after leaving the center. Comparisons of patients against their non-user friends, who achieved career success, married, and had children, on top of work stress, and personal relationship issues were viewed as driving factors for substance relapse.

There were two personal factors (*lack of willpower* and *mental health issues*) cited exclusively by patients as reasons for substance relapse. The *lack of willpower* was perceived by seven patients as reasons for relapse as they realized that they were lacking mental strength and fooled themselves in believing they could overcome substance use easily. For 1 patient, *mental health issue* was a trigger for relapse as he suffered from depression, which was a result of family disputes and broken family relationships. There were three environmental factors (*easy substance accessibility*, *parental rejection*, and *methadone replacement therapy*) cited as substance relapse factors. A patient reported that most substance sources were peers within the neighborhood and, thus, it was difficult to avoid the temptation to buy and use substances again. *Parental rejection* was also cited by a patient as a harsh reality when patients were attempting to start anew post-treatment, leading to relapse episodes to cope with sadness and disappointment. There were mixed responses toward the use of *methadone replacement therapy* to reduce usage and dependency on opioids. While a patient reported that methadone replacement therapy was an effective method to reduce dependency toward heroin, another patient craved for higher dosages of methadone instead of the normal substance used.

#### Motivation to Seek Treatment

As seen in Table 2, the impact of substance use on the self (*financial constraints*), family (*inability to support aging parents* and *neglected responsibilities*), and work and health (*physical deterioration* and *emotional torture*) was pivotal in motivating patients to seek treatment. Due to the costly price of substances, two patients reported being unable to finance their habit. Moreover, the constant need to seek monetary funds was physically and emotionally draining. A patient realized that his substance use habit affected the ability to support his aging parents, as the money that was allocated for household needs and health care was used to buy illicit substances. Another patient realized that the effects of substance use were making him neglect family responsibilities. A patient cited the *impact on work* as a reason to seek treatment, as he found that the effects of substance use impaired his ability to function at the work place, which was a cause of many family disputes. The experience of *deteriorating physical health* was reportedly motivated two patients to seek treatment, while another patient could no longer bear the *constant mental torture* felt during substance use and withdrawal phases. There were 19 patients who stopped substance use because they were tired and disappointed with the past way of living and hoped for a better future, while 14 patients entered treatment under court orders without the intention of stopping substance use.

#### Program Evaluation

As seen in Table 3, four activities were identified by patients and rehab staff as favorite treatment components: *spiritual studies* (13 references), *vocational workshops* (12 references), *counseling* (10 references), and *recreational activities* (8 references). Spiritual studies, either religious-based or civic-focused teachings, were viewed as essential toward helping patients resolve personal issues and bring about internal changes. Vocational skill components were beneficial toward preparing patients to enter the workforce after treatment completion. Group counseling sessions were able to provide patients with the opportunity to share their problems and resolve it as a team, while individual counseling with professionals help patients resolve personal issues that are confidential in nature. Recreational activities were also a favorite, as it did not require special skills and helped improve patients’ physical health.

**Table 3:** **Treatment program evaluation from rehab patients and staff perspective**.

Categories	Themes	Sub-themes	Quotes
Favorite components	Vocational workshops		*“I like the vocational aspects of the program such the arts and craft workshop. They let us know the value of skill in the market so that we can support ourselves after the program.” [GP05]*
			*“…for those who are unemployed, the program tries to link the skills taught with relevant jobs which they can consider venturing into.” [GP12]*
			*“The vocational component is also important for clients after they leave the center.” [GS03]*
	Spiritual studies		*“…the spiritual part…treated my senses. This program also answered some of the fundamental questions that I always thought of. Previously I was filled with a lot of anger, depression and negative thoughts. Even when others treated me kindly, I thought that there was a motive behind it or they are out to get me. Through this session, I learnt about love, kindness and how to think positively.” [PP02]*
			*I think spiritual studies bring the most internal changes in the students.” [PS02]*
			*“I feel that programs with a religious or civic focused component would be important to improve character. For example, the Muslim clients can attend religious classes while non-Muslim clients can go for Moral Education lectures.” [GS03]*
	Counseling		*“The group counseling sessions allow me to interact with friends having the same problems as me. Also the supervisors here are also very encouraging and friendly.” [GP04]*
			*“I like that it gives me the opportunity to listen and learn from others’ problems. Finding the solutions with the help of others also helps take out the frustration of resolving something that I can’t get around.” [GP09]*
			*“The rehab program…was based on the didactic approach. There were…individual and group counselling. I feel that the combination of components was ok and suits the type of clients in the centre…” [GS03]*
	Recreational activities		*“I like the recreational component where I can get some exercise.” [PP04]*
			*“I am not really skilled in any area but I like recreational activities the most.” [PP11]*
			*“…sports activities are…to maintain their physical fitness.” [PS05]*
Limitations	Limited and unsafe infrastructure		*“…the grounds are quite cramped. There are no proper places to do outdoor exercise. When drug addicts newly enter the center, they have not overcome their drug problem and experience withdrawal symptoms in the first two weeks. However, no medical facility is conveniently available within the grounds to help them overcome drug withdrawal. Currently, we have to fork out our own money to go to a private clinic.” [GP01]*
			*“…staff offices are often crammed with little space for all the paperwork. The staff also face a risk when dealing with drug addicts who just arrived at the center as we are mostly unaware if they are suffering from illnesses such as tuberculosis or HIV. The diagnosis will only be known after the medical unit at the rehab center has done a medical examination.” [GS02]*
			*“…the rehab center building was quite old and the location of the counselling center was quite dangerous. It was situated at the bottom of a hill, which can be dangerous if there were landslides.” [GS03]*
	Curricular issues	Limited activity range	*“It is a bit boring during the rest hours as there is nothing interesting to do other than sleep. There are no suitable indoor activities either because I don’t know how to play carom, which is the only game choice currently.” [GP02]*
			*“It can be boring but provides a semblance of normalcy as normal people also have routines like waking up and going to work.” [PP06]*
		National unity values	*“There is insufficient content about national unity taught in the center. Although the inmates are united within the center regardless of race and ethnicity, there exists a racial gap when they leave the center and re-enter society.” [GS05]*
		Medium of instruction	*“I find the spiritual studies a little bit difficult to understand because of the language medium.” [PP14]*
			*“At the moment, the classes are conducted in English with Chinese translation. Due to my limited capability in the English language, I sometimes have trouble keeping up. I can only catch bits and pieces of it.” [PP15]*
		Limited job links	*“I feel the skills taught in the workshop can only be used at the center as there is no job network established that can help link the skills and proper jobs outside.” [GP07]*
	No support network		*“There is no way to keep in touch with friends at the center to keep tabs on how they are doing and forming a support network.” [GP07]*
	Insufficient treatment practitioners		*“There is a lack of resources and staff, especially in regards to security. There is a huge area within the center but there is too few staff to cover all areas.” [GS02]*
			*There was a rotation system for counselling sessions due to insufficient counselling staff. Therefore, rather than continuous sessions, some clients are seen only once a month.” [GS04]*
Suggested improvements	Tailored approach		*“…it would be more effective if the program could be tailored to different types of cases. For example, old cases where inmates had been in and out of several rehab centers should be separated and dealt with differently from new cases with first timers. This is because for old cases, the inmates pretty much about the ongoing of the program while for first timers, they may only be able to accept 30% of what is taught in rehab.” [GS02]*
			*“Improvements to the treatment process must be done to ensure the rehab sessions meet the needs of the client. They should evaluate what the clients require, whether at the prison stage or at the agency level before the clients are distributed to different rehab centers based on the categories. For instance, some centers are more religious based while others practice community therapy, psychosocial approach and other approaches.” [GS03]*
	Treatment facility upgrade		*“…have a clinic within the center for patients undergoing methadone therapy. At the moment, it is expensive to go to a private clinic monthly for treatment as they charge RM 13 every time.” [GP07]*
			*“The weather these days are really hazy and hot. Moreover, the location of the center is just next to the road and the air is really dusty. It would be nice if the sleeping quarters for the residents could be air-conditioned.” [GP15]*
			*“…the center is currently working on upgrading the security of the center such as building stronger gates to prevent new drug addicts from escaping. This will better allow us to control the ins and outs of visitors and residents within the center compound.” [PS05]*
	Improved curriculum	Expansion of activities and services	*“…Probably, they can consider more age-appropriate activities for elderly drug addicts.” [GP02]*
			*“Having a garden in which fruits and vegetables can be planted would be beneficial for the center’s sustenance and for selling.” [GP06]*
		Provision of job links	*“…the center can introduce places of work that we can go after graduation. This is important because we need to be able to find a source of living after rehab. At the moment, we are left on our own once we leave the center. So, there is a sense of discontinuity.” [PP13]*
			*“It would be good if more can be done about work opportunities for former drug addicts…some employers are wary about taking them as employees because credibility may be affected when there is a constant change in employees.” [GS02]*
			*“There is a need to improve occupational opportunities for inmates or clients who have completed rehab. For example, there has been a joint venture with MARA to absorb clients into workshops but so far, this has only been done with the Sepang rehab center. In fact, all centers require joint ventures such as this.” [GS05]*
	Group relationship enhancement		*“I think more efforts need to be done to strengthen the relationship between members undergoing the same program.” [GP13]*
			*“…there is a need for more interaction between the students or inmates and leaders to learn more about them as an individual.” [PP02]*
	Improved discipline		*“Initially, I felt a little suffering because of the loss of freedom.” [PP15]*
			*“Naturally, there should also be punishment when needed so that they are aware of the consequences of their actions.” [GS02]*
			*“All clients or patients must follow the each program strictly.” [GS03]*
	Improving staff management		*“Besides studying the results of reports, the upper management needs to take time to go down to level of the inmates and rehab staff to understand the real problems faced in treatment and rehab…there should be an increase of religious officers and counselors placed in the center. The current ratio is 1 counsellor for every 500 inmates. Besides that, not many officers are committed.” [GS04]*
	Increased voluntary admissions		*“I feel that the program will be more effective if the person enters voluntarily.” [GP01]*

There were various limitations highlighted by patients and staff: *curricular issues* (nine references), *limited and unsafe infrastructure* (eight references), *insufficient treatment practitioners* (three references), and *no support network* (one reference). *Curricular issues* that posed an issue to patients and staff include limited activity range, lack of national unity values, problems with medium of instruction, and limited job links. In the government center, two in-patients had trouble finding indoor activities that were age-appropriate to fill time after completing treatment activities. Alternately, a private patient found the treatment schedule to be rather conventional yet provided a sense of normalcy. Due to the multi-ethnic background of patients, some patients in the government and private center had trouble understanding and communicating when a standard language was used in teaching components, such as spiritual studies or group counseling. A staff also noted a lack of content related to national unity in the curriculum, which was an important aspect in bridging the re-integration process. Although vocational skills were taught at the centers, the patients felt that there was limited opportunity to transfer learnt skills as no job links were provided to aid the transition into working members of society.

*Limited and unsafe infrastructure* was also a major concern for staff and patients. Limited on-site medical facilities for patients were an inconvenience to two patients under methadone replacement therapy. A staff voiced concern over the risk of contracting infectious diseases since patients were often admitted into rehab without proper clinical checks. Additionally, limited space to conduct rehabilitative activities, the lack of working space within the center and the risky location of the center were also cited by four patients and staff as factors that affect treatment and work satisfaction. Most patients also felt disjointed upon leaving the center, as there was *no social network* to remain connected with their rehab peers to provide mutual support. Three staff raised the issue of having *limited rehab professionals* catering to the needs of many patients. Moreover, the security of the center was compromised with insufficient staff to patrol the grounds.

There were seven important themes generated in regard to suggestions for improving rehab programs: creating a *tailored approach to treatment*, *upgrading treatment facilities*, developing an *improved curriculum*, *enhancing group relationship*, *improving staff management*, *improving patient discipline*, and *increasing voluntary admissions*. Five staff were aware of the need to apply a *tailored approach to treatment* by treating each patient according to individual circumstances. This includes designing different treatment plans for old and new substance users, assessing patient needs before treatment admission and providing specialized counseling to patients facing discrimination and stigmatization, such as LGBTs and HIV patients. *Upgrading treatment facilities* was strongly recommended by four patients and two staff to provide a more comfortable environment for patients, such as cheaper on-site medical services, stronger security, and upgraded accommodation.

With respect to developing an *improved curriculum*, expansion of activities that were age-appropriate and could teach patients self-sustenance was suggested by a patient. In addition three patients and two staff suggested providing patients with the opportunity to intern with prospective employers, build job networks, and obtain good job references. Efforts in *enhancing the group relationship* through interaction between patients of different cultures were suggested by two patients. A patient felt that through *increased voluntary admissions*, the creation of positive relationships with patients who were self-motivated to overcome substance addiction would be beneficial toward their peers’ goal to reduce substance use. Four staff provided suggestions to *improve staff management*, such as the adoption of a bottom-up approach by upper management to better understand the daily issues faced by patients and treatment providers and finding a solution to high turnover rates by implementing stricter recruitment criteria to ensure that only committed staff are employed. Opportunities for staff development should also be provided to committed staff lacking training in certain areas. There were contradictory opinions between patients and staff about *improving patient discipline* due to different treatment approach in the government and private center. Three patients in the private center felt stifled by the strict routines and suggested having a lightened schedule with sufficient time to relax in between. However, a staff in the government center felt that there should be stricter action and consequences related to treatment compliance and patient conduct.

## Discussion

To recap, this study was designed to examine substance abuse and relapse from the perspectives of rehab patients and staff. Conjointly, feedback from patients and staff about the quality of substance treatment services in Malaysia was obtained. One of the focal points of this study was to investigate the prevalence of substance use progression in this patients sample and the conditions of patients and their peers’ family relationships. Based on our findings, most patients were poly-substance users and did not demonstrate a progression from soft to hard drugs, which was congruent with past studies (10, 11). The findings indicated that 25 patients in this sample and their peers did not conform to past research findings (14, 15), as they experienced normal levels of relationship with their parents and family members. These patients did not conform to the stereotype of being from troubled family backgrounds and low socioeconomic status. Instead, these patients were from families in which either one or both parents were working and, thus, never experienced any form of economic deprivation. Changes in substance user demographics has been occurring over the decades with more users from white collar workers, civil servants, and college student populations, in addition to children as young as 12 years (45).

The levels of assertiveness against substances that would be exhibited by patients at the point of treatment were also investigated. Most patients felt that were able to be assertive and resist offer of substances from friends or strangers in social situations. This finding can be construed as a positive implication of the effectiveness of treatment programs practiced in both rehab centers despite its various limitations. We were notably interested to examine the themes that would emerge from patient and staff interviews about substance use, relapse factors, and motivation to seek treatment. The thematic analysis yielded informative findings on substance use factors, whereby the significant influence of external factors, such as peer influence and family conflicts as well as personal factors like curiosity, enjoyment, tension release, relationship issues, health issues, and unemployment were evident in both staff and patients’ responses. While the influence of external factors, such as family conflict and peer influence, on substance use has been widely researched in past studies (16, 18, 19), the responses here also indicated, in accord with some previous writings (46), that youths use illegal substances for many different reasons: some use to cope with stress or when they must study long hours. Some turn to substance use simply out of curiosity, while others see substance use as a means of socializing (46). Similar to what has become quite a serious problem in the United States (1), some Malaysians also reported becoming addicted to substances (such as pain killers) originally prescribed due to health issues.

Findings on relapse factors were more congruent with Scorzelli’s study (25) in which personality correlates like depression (mental health) and environmental factors, such as family dysfunctions and old peer influence, were cited by patients and staff. Additional findings that were of interest include personal factors, such as the lack of willpower and external factors such as easy substance accessibility (47) and rejection from parents (48). There were contradictory findings on the influence of methadone replacement therapy, which was reported by some patients as a cause for relapse, when its original intent was to reduce patients’ dependency on opiates.

Findings on patients’ motivation for seeking treatment support results from past studies (27, 28), indicating that self-motivation to change is instilled when users experience an influential impact on the self, family, work, and health. The responses also highlighted a need to promote voluntary admission into rehab, as most rehab patients were mostly present due to court orders. Thus, barriers toward treatment, such as fear of stigmatization, prosecution, and imprisonment, in addition to impact on employment must be urgently addressed (49). Nevertheless, it was encouraging that most patients who entered treatment under court orders with no intention to stop substance use eventually found their goals to change and worked toward it.

The statistical analysis indicated that there were no significant differences in treatment satisfaction scores between patients and staff, although satisfaction ratings accorded by patients were lower than the rehab staff. Caution should be practiced when interpreting results related to patient satisfaction and evaluation. While this result could be interpreted as patients’ expectations toward the program were met by treatment services and outcomes envisioned by the rehab staff (50), the possibility that staff evaluation were liable to biases should not be discarded. Based on patients’ feedback, it was acknowledged that patient satisfaction evaluation is not commonly practiced. This could be due to doubts due to possible threats on the professional interests (status, livelihood, and standards) of treatment providers (51).

Lastly, we were interested to obtain quantitative feedback from patients about their feelings and emotions in their most recent treatment session. Qualitative feedback was also obtained from patients and staff on treatment components that were most favorable, its limitations and suggestions for improving treatment effectiveness. Although the patients provided mostly positive feedback about the program in terms of depth in content, smoothness in progress of each treatment session, improvements in levels of positivity, and empowerment in working toward treatment goals, the views of the minority in raising issues that should be promptly remedied must not be neglected. These issues include the inability to address all personal issues within a group session, cancelation of allocated sessions due to the non-presence of treatment provider and the inability to cater to the needs of elderly patients. In such cases, more efforts should be invested on offering individual counseling to patients with problems that require further expertise and time, arranging a secondary treatment provider when the primary provider is unable to attend the sessions and creating activities that would appeal to rehab patients from the older age group.

Qualitative responses from the program evaluation suggest that the patients enjoyed a holistic approach to treatment involving a combination of cognitive–behavioral, medical, social, and spiritual components (52). Responses related to program limitations and suggested improvements revealed a consensus between patients and staff that a tailored approach to treatment should be developed and practiced. For instance, various personal and environmental factors within each individual case should be considered when creating treatment plans, such as family relationships, peer networks, employment conditions, as well as physical and mental health issues. These factors can greatly influence the treatment approach used to provide optimum treatment outcomes. Additionally, treatment facilities should be upgraded with ample space for patients to conduct treatment activities and for staff to work comfortably. An improved curriculum with more emphasis on national unity and addressing the needs of special groups, such as the elderly and patients with medical conditions, such as HIV and tuberculosis, was also suggested.

The patients also highlighted the need to enhance group relationships. Besides providing patients a platform to share, discuss, and resolve issues that may impede treatment as a group, more efforts to improve group cohesiveness is needed. Icebreakers and trust building activities are useful to help patients feel comfortable to disclose their problems, while developing mutual understanding, respect, and empathy. The bonds developed during treatment sessions could extend after treatment sessions or completion, resulting in the formation of a strong social support network for life. There was also an urgent need to improve staff management and address the issue of employing more trained personnel to cater to the needs of large groups of patients. The contradictory findings in regard to improving patient discipline suggests that treatment providers need to ensure that treatment programs are developed to provide patients with a balanced amount of activities to help them learn and resolve substance issues, with sufficient time to rest and recuperate.

The mixed method approach that was applied in this study is an established research method that allowed the exploration of a broad range of questions without being solely restricted by the quantitative or qualitative research paradigm. The convergence and corroboration of research findings across multiple perspectives (i.e., patients and treatment providers) on the topic of substance abuse provided stronger concluding evidence (53) about substance abuse and relapse factors as well as motivations to seek treatment. A limitation of this research lies in its small sample size, which was essentially unavoidable due to the time-intensive nature of qualitative aspects in this research (53). For this reason, it is inadvisable to generalize too much to the broader population. Despite this limitation, the analyses of patients and staff experiences and beliefs did provide several unique insights that can guide future research. For example, this study suggests that several important environmental and personal factors play an important role in the long-term success or failure of rehabilitation efforts. Future research could look at evaluating these factors in patients and using them to tailor individual treatment programs. In addition, several patients pointed out a dearth of post-treatment support. This may be an important area of focus for future intervention research: how can we best provide peer and social support that will make patients least likely to revert to previous bad habits at post-treatment? Additionally, with the aim of improving prevention and education programs aimed at the general public, future studies could examine substance abuse and relapse from the perspective of non-user populations. By comparing lay perceptions with actual users’ experiences, we might be able to target specific lay misperceptions and, thus, improve prevention efforts. Another area that was not explored in this study was differences in user profiles and substance use history between rehab patients from rural and urban areas. Due to the recent increase in migration from rural areas in Malaysia to more urbanized regions, such as Selangor, we were able to collect data from a diverse range of rehab patients, including some from East Malaysia (Borneo). In future studies, it would be of interest to investigate how issues in rural communities, such as poverty, unemployment, and isolation, contribute to drug use in comparison with urban areas. The difference in access to prevention and treatment services in rural areas (54) also warrants further study. For example, technology and long-distance interactive instructional materials for prevention education will become increasingly useful (55, 56). Thus, the implementation of computer-mediated communication (CMC) toward educating children and youths in geographically disadvantaged areas should be further explored.

It can be concluded from this study that various improvements are needed in the area of substance abuse treatment services in Malaysia, especially in adopting a tailored approach to treatment, improving the group relationship between patients as well as patient–provider relationship, in addition to upgrading treatment facilities and space. Improvements in program curriculum are needed, which includes expanding the range and age-appropriateness of certain treatment activities as well as expanding existing after-care services to include post-treatments follow-ups. Moreover, assessment of treatment evaluation from the perspective of staff and patients should be considered for routine practice as a way for treatment providers to ensure that treatment goals, expectations, and outcomes between patients and staff are aligned. Research findings regarding program evaluation and patient satisfaction are instrumental toward developing a systematic system to manage rehab patient data and monitoring rehabilitative progress. On a national level, these findings may be useful toward building substance treatment policies such as making it compulsory to conduct needs and patient background assessments before treatment placements since rehab centers in Malaysia offer different treatment specialties. Practicing a more inclusive approach to treatment with consideration for input from patients about their individual progress is also an essential step in moving treatment styles toward a tailored approach.

## Author Contributions

QC contributed towards the research conception and design, data collection, data analysis and interpretation, writing of the article draft, and critical revisions. CT and GB participated in the research conception and design, data collection and critical revision of the article draft. HM and RK contributed towards the final critical revision of the article.

## Conflict of Interest Statement

The authors declare that the research was conducted in the absence of any commercial or financial relationships that could be construed as a potential conflict of interest.
